# Cholesteric mesophase based 1D photonic materials from self-assembly of liquid crystalline block and random terpolymers containing chromonic molecules†

**DOI:** 10.1039/d1ra00899d

**Published:** 2021-04-20

**Authors:** Reuben Bosire, Dennis Ndaya, Rajeswari M. Kasi

**Affiliations:** Department of Chemistry, University of Connecticut Storrs CT 06269 USA rajeswari.kasi@uconn.edu; Polymer Program, Institute of Material Science, University of Connecticut Storrs CT 06269 USA

## Abstract

We describe the influence of competing self-organizing phenomena on the formation of cholesteric mesophase in liquid crystalline brush block terpolymers (LCBBTs) and liquid crystalline random brush terpolymers (LCRBTs) containing chromonic molecules. A library of LCBBTs and LCRBTs are synthesized using ring-opening metathesis polymerization (ROMP) of norbornene side-chain functionalized monomers comprising cholesteryl mesogen (NBCh_9_), chromonic xanthenone (NBXan), and poly(ethylene glycol) (NBMPEG). Compression molded films of LCRBTs containing chromonic molecules display multilevel hierarchical structure in which cholesteric mesophase co-exists with π–π stacking of the chromonic mesophase along with PEG microphase segregated domains. This is unexpected as conventional LCBCPs and LCBBCs that lack chromonic molecules do not form cholesteric mesophases. The presence of π–π interactions modifies the interface at the IMDS so that both chromonic and cholesteric mesophases coexist leading to the manifestation of cholesteric phase for the first time within block architecture and is very reminiscent of previously published LCRBCs without chromonic molecules. The key to the observed hierarchical assembly in these LCBBTs containing chromonic molecules lies in the interplay of LC order, chromonic π–π stacking, PEG side chain microphase segregation, and their supramolecular cooperative motion. This unique “single component” polymer scaffold transforms our capacity to attain nanoscale hierarchies and optical properties from block architecture similar to nanoscale mesophases resulting in random architecture.

## Abstract 2

We describe the influence of competing self-organizing phenomena on the formation of cholesteric mesophase in liquid crystalline brush block terpolymers (LCBBTs) and liquid crystalline random brush terpolymers (LCRBTs) containing chromonic molecules.

## Graphical abstract

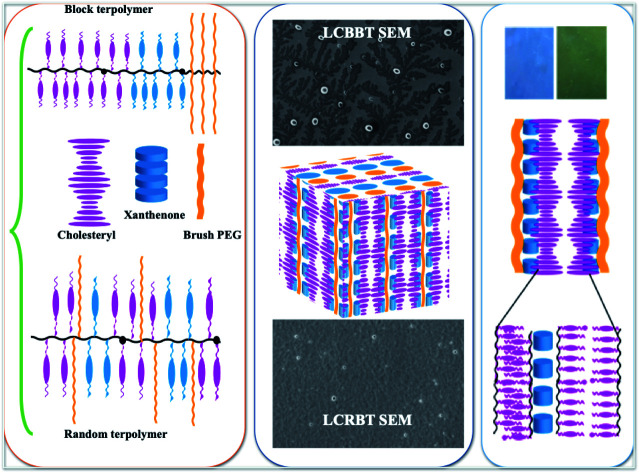

## Introduction

Polymer based 1D photonic nanostructures with domain sizes comparable to the wavelength of light are formed by using (i) small molecular additives to swell domains of linear block copolymers (BCPs),^1–3^ (ii) self-assembling ultra-high molecular weight brush block copolymers (Brush BCPs),^4–6^ or (iii) 1D photonic domains from cholesteric (N*) liquid crystalline random copolymers (LCPs).^7–10^ However, cholesteric mesophases and their consequent stimuli-responsive liquid crystalline properties have never been observed using block copolymers (BCPs), brush block copolymers (Brush BCPs), liquid crystalline block copolymers (LCBCPs) or liquid crystalline brush block copolymers (LCBBCs).^11^ In this work, we demonstrate, for the first time, a singular approach to self-assembly of cholesteric mesophases from liquid crystalline block copolymers.

Block copolymers undergo microphase separation due to unfavorable mixing enthalpy and weak mixing entropy.^12^ Microphase segregation offers opportunities for preparation of materials with tunable nanostructured morphologies including lamellar, cylindrical, bicontinuous, and spherical morphologies although typically accessible domain sizes for commonly studied BCPs lie between 10–100 nm.^13–15^ To introduce another level of ordering, liquid crystalline block copolymers (LCBCPs) display order on 3–10 nm length scales associated with the formation of LC ordering and on larger length scale from 10 to 100 nm associated with the microphase separation of the blocks.^11^ To improve processability, alignment and ordering kinetics, LCBBCs scaffolds comprising side-chain LC mesogens in one block and brush-like moieties in the other block are synthesized. The LCBBCs show interesting features due to (i) supramolecular cooperative assembly of liquid crystalline mesophase and block copolymer microphase segregation,^16,17^ (ii) low entanglement and fast assembly kinetics of brush molecules,^18,19^ and (iii) ability to tailor block copolymer order-disorder temperature and liquid crystalline transition temperature.^18,19^ This LCBBC platform self-assembles into a hierarchical structure with LC order in 3–10 nm range, brush BCP ordering due to phase segregation of brush molecules in the 10–200 nm range. Despite the ease of synthesis, processing and self-assembly of BCP, LCBCP and LCBBC scaffolds, periodicities corresponding to wavelengths in the visible range with the cholesteric mesophases has been particularly elusive.

The role of microphase segregation of the non-LC block on LC mesophase behavior in chiral and non-chiral LCBCPs and LCBBCs has been investigated. The microphase segregation even in chiral LCBCPs and LCBBCs unwinds pitch of helical mesophases due to presence of preferred anchoring condition at the IMDS of microphase segregated domains. Thus, cholesteric mesophases are not observed in these non-chiral and chiral platforms.^16^ One may envision that the development of LCBBC with additional supramolecular interactions including H-bonding or π–π stacking to produce hierarchical mesophases and co-exiting morphologies that are not generally observed in conventional platforms. We question if molecular interactions, especially resulting from π–π stacked aromatic cores of chromonic molecules,^20^ could potentially serve as a method to enhance cooperative LC–LC interactions between the chromonic molecules and LC molecules within a block scaffold architecture? Furthermore, we also question whether LCBBCs containing these chromonic molecules will form functional, stimuli-responsive, cholesteric mesophases that are easily produced from linear and branched liquid crystalline random copolymers?

Lyotropic chromonic liquid crystals (LCLCs) are a unique subset of lyotropic LCs possessing rigid, plank-shaped molecules with aromatic cores and often functionalized at the periphery with ionic groups to aid solubility in aqueous solutions.^21,22^ These molecules tend to stack face to face into aggregates aided by strong π–π interactions between aromatic cores and nanophase separation with hydrophobic cores surrounding groups.^23^ These molecules include a variety of drugs and dye molecules self-organize into chromonic π–π mesophases in water.^24,25^ Upon removal of water, dried films of π–π stacked chromonic liquid crystals are produced for production of masks, templates, biosensing and optical applications.^26^

Herein, we report the design of polynorbornene scaffold comprising ABC type block architecture wherein A block contains side liquid crystalline cholesteryl molecules, B block contain chromonic xanthenone molecules, and C block containing brush-like PEG molecules that serves an internal plasticizer. This new liquid crystalline brush block terpolymers (LCBBTs) scaffold comprising chromonic xanthenone self-assembles into co-existing cholesteric mesophase, π–π stacked chromonic mesophase, and microphase segregated PEG domains. Although helical multilevel hierarchical materials has been demonstrated using chiral polypeptides, BCPs and chiral PS-PLLA block copolymers, to the best of our knowledge cholesteric mesophase in conjunction with microphase segregated domain has never been observed from BCP architecture. The key to the observed hierarchical assembly and controlled structure formation lies in the interplay of LC order, chromonic π–π stacking, PEG side chain microphase segregation, and their supramolecular cooperative motion. This unique “single component” polymer scaffold transforms our capacity to attain nanoscale hierarchies from block architecture similar to nanoscale mesophases resulting random architecture.

## Synthesis of monomers

Monomer, 5-{9-(cholesteryloxycarbonyl)nonyloxycarbonyl}bicyclo[2.2.1]hept-2-ene (NBCh_9_) is synthesized according to previously reported procedure. Poly(ethylene glycol)monomethyl ether functionalized norbornene (NBMPEG, *M*_n_ of MPEG = 2000 g mol^−1^) is synthesized from modified literature procedure.^27^ The monomer bearing xanthenone (NBXan) is synthesized (Scheme S1†) and its purity confirmed by ^1^H NMR and MS (Fig. S1†).

## Preparation of homopolymers

The synthesis of homopolymers P(NBMPEG) and P(NBCh_9_) has been previously synthesized by established protocols. Polynorbornenes containing xanthenones, P(NBXan), are synthesized by adding monomer NBXan (500 mg) into a 100 mL round bottomed flask, capped and purged with nitrogen gas. In a separate vial, modified Grubbs catalyst is dissolved in CH_2_Cl_2_, purged with nitrogen, transferred into the flask containing the monomer. The flask is purged again with nitrogen. The rate of homopolymerization for the new monomer is monitored *via*^1^H NMR (Fig. S2†) is similar to that of PNBCh_9_ (ref. 10) and PNBMPEG.^27^ Quantitative conversion of NBXan to PNBXan is achieved in less than 10 minutes.

## Preparation of terpolymers

**Fig. 1:**
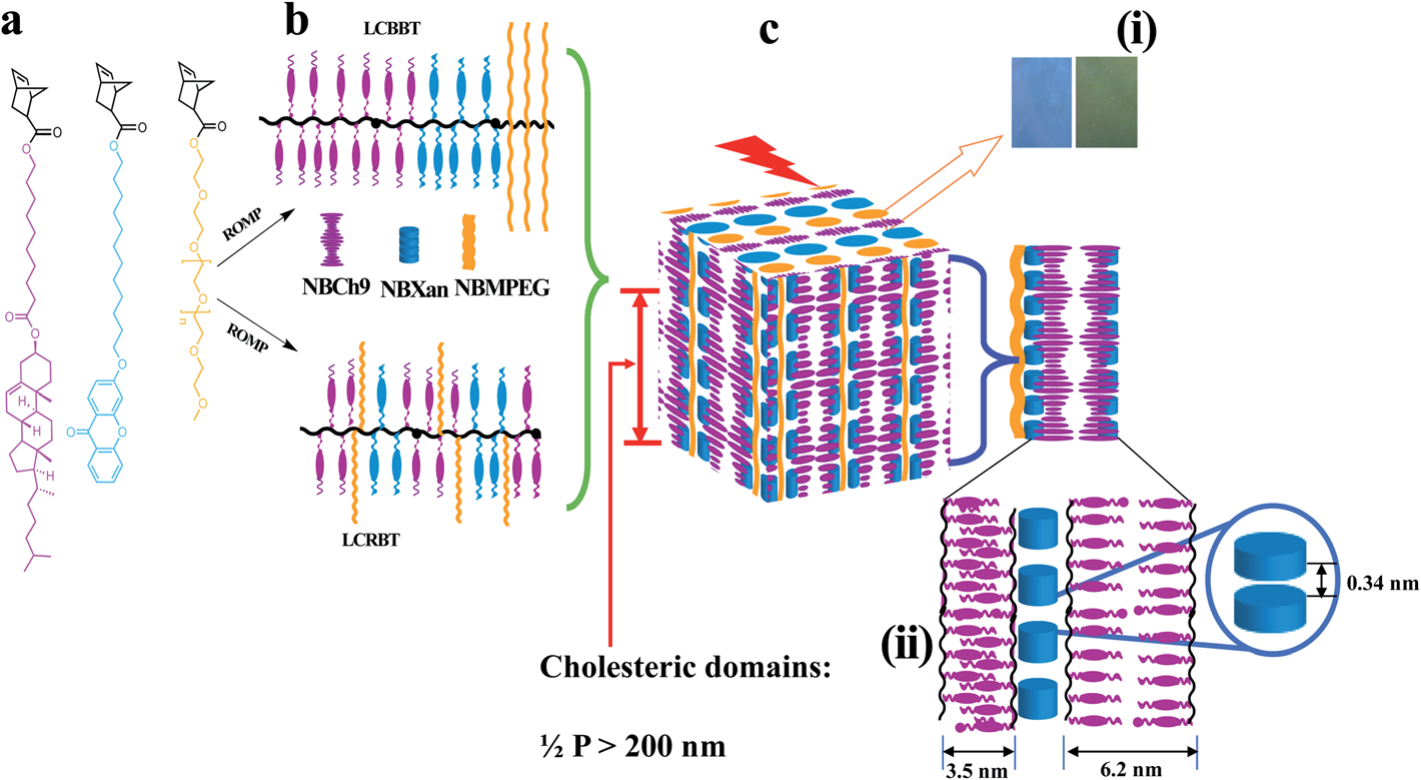
Schematic illustration for 1D photonic material based on cholesteric–chromonic interactions lacking long range order: (a) monomers (NBCh_9_, NBXan and NBMPEG), (b) synthesis of random and block terpolymers by ROMP using modified second generation Grubbs catalyst, and (c) illustration of hierarchically self-assembled 1D photonic material. (i) Photonic properties originating from Ch* mesophase (300–600 nm) from compression molded TPX75B and TPX75R films with blue and green color reflections respectively. (ii) Molecular packing for the film samples comprising hierarchical structure of MPEG domains (domain size, *d* = 13.2 nm), individual π–π stacking of xanthenone molecules (3.4 Å or 0.34 nm), and polymorphic smectic domains arising from cholesterol majority matrix in all samples, monolayer smectic layers (∼3.5 nm) and smectic bilayer (∼6.1 nm) as shown in Table S2.†

A series of random and block terpolymers bearing NBCh_9_, NBXan and NBMPEG are prepared by ROMP using modified Grubbs catalyst second generation ((H_2_IMes)(pyr)_2_(Cl)_2_RuCHPh) with CH_2_Cl_2_ as a solvent. In a typical example block terpolymers (TPX75B) is prepared by dissolving 522.8 mg of NBCh_9_ in CH_2_Cl_2_, followed by purging with nitrogen gas, followed by addition of 9.9 mg of modified Grubbs catalyst dissolved in CH_2_Cl_2_ is added and purged with nitrogen. In the next step, NBXan (70.0 mg) in CH_2_Cl_2_ and NBMPEG (102.5 mg) in CH_2_Cl_2_ respectively, are each allowed to polymerize for 30 minutes before being terminated by addition of ethyl vinyl ether and precipitated into methanol before being dried overnight. The random terpolymers are made in a similar manner except that all the monomers are mixed in one step before adding the catalyst (Fig. 1). The block and random terpolymers are then characterized by ^1^H NMR and GPC as shown in Table 1.

**Table 1:** Polymer composition and molecular weight characterization

Entry^a^	Polymer description	Weight percentage^b^ (from ^1^HNMR)			*M*_n_ c kDa mol^−1^ (*Đ*_M_)
		NBCh_9_	NBXan	NBPEG	
TPX75B	P(NBCh_9_-*b*-NBXan-*b*-NBPEG)	75.0	9.7	15.3	44.8 (1.15)
TPX85B	P(NBCh_9_-*b*-NBXan-*b*-NBPEG)	84.9	9.7	5.4	41.4 (1.21)
TPX75R	P(NBCh_9_-*b*-NBXan-*b*-NBPEG)	75.7	10.0	14.3	45.7 (1.09)
TPX85R	P(NBCh_9_-*b*-NBXan-*b*-NBPEG)	84.5	11.0	4.5	50.0 (1.10)
*HPX100	PNBXan	—	100.0	—	23.9 (1.03)

a For purposes of consistency and clarity the terpolymers are denoted by TPX_*y*_B or TPX_*y*_R where *y* is the target theoretical weight percent composition of NBCh_9_, B denotes block copolymer and R denotes random copolymer HP = homopolymer, TP = terpolymer, X= xanthenone.

b Weight percentage of each monomer in random terpolymer and copolymer samples are determined by ^1^HNMR integrations of the peaks at 4.6, 3.36 and 7.66 ppm corresponding to NBCh_9_, NBMPEG and NBXan monomers, respectively.

c Determined by GPC with ELSD detector, where THF was used as eluent and polystyrene (PS) standards were used to construct a conventional calibration. *HPX is polyxanthenone homopolymer.

A representative ^1^H NMR spectrum of TPX75B is shown in Fig. S2.† By comparing the integration of the peaks at 3.36, 4.6 and 7.66 ppm, corresponding to NBMPEG, NBCh_9_ and NBXan, wt% of each monomer is determined in block and random terpolymers. Table 1 summarizes molecular characterization for the block terpolymers, random terpolymers, and homopolymer (HPX100).

## Thermal properties

Thermogravimetric analysis (Fig. S4†) indicates that LCRBC and LCBBC terpolymers in all compositions investigated shows 5% sample weight loss around 350 °C under nitrogen at a heating rate of 20 °C min^−1^.

The thermal transitions of the terpolymers are investigated by differential scanning calorimetry (DSC). Samples are initially heated to 150 °C, cooled to −50 °C and reheated to 150 °C at a scan rate of 10 °C min^−1^. The transition temperatures from the first cooling cycle and corresponding enthalpy changes (Δ*H*) are established this way and are tabulated in Table S1.†

TPX75B (block terpolymer with 75 wt% of NBCh_9_) shows two LC transitions at 80.7 °C and 100.9 °C and PEG crystallization temperature at −34.5 °C. TPX85B (block terpolymer with 85 wt% of NBCh_9_) shows a similar trend, Table 1. Both TPX75B and TPX85B show depressed PEG crystallization temperature, which is thought to originate from confinement effects.^28^ This is in contrast to LCBBC78,^27^ which is a diblock copolymer of NBCh_9_ (78 wt%) and PEG brush unit (22 wt%), and shows two LC transitions and backbone polynorbornene glass transition temperature at 85.2 °C and 105.7 °C, and 23.5 °C, respectively, but does not present any nanoconfined PEG crystallization temperatures. In sharp contrast to TPX75B, only one LC transition at 83.5 °C for terpolymer with random architecture and 75 wt% of Ch_9_, TPX75R, and 87.7 °C for random terpolymer and 85 wt% of Ch_9_, TPX85R, is observed. Both these random terpolymers show PEG crystallization temperatures ranging from −21 and −43 °C due to nanoconfinement effects.

**Fig. 2:**
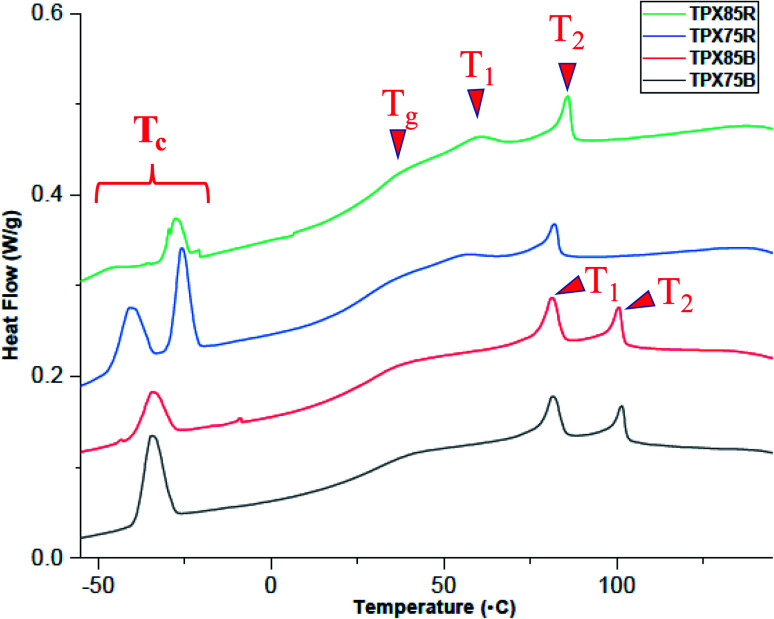
DSC curves showing first cooling trace of random and block terpolymers with PEG crystallization (*T*_c_) glass transition (*T*_g_) and LC mesophase transitions (*T*_1_ and *T*_2_). *T*_2_ is the LC clearing temperature and generally glass transition temperature and LC mesophase transition of terpolymers lies between 26–37 °C and 80.7–100.9 °C, respectively. A heating/cooling rate of 10 °C min^−1^ is used in this study.

For all block and random terpolymers in this study, the PEG crystallization temperature (*T*_c_) and associated enthalpy of cooling are lower than polynorbornene containing PEG brushes (PNBPEG) due to the dilution of PEG chains within polynorbornene matrix (Fig. 2). Two different populations of crystalline regions from PEG in random architectures corresponding to the two peaks in the region of −21 °C and −43 °C are observed. However, only one significant population of crystalline regions from PEG corresponding to one peak in the region of −34.48 °C for TPX75B and −33.70 °C for TPX785B is observed. The polynorbornene backbone glass transition temperature of both random and block systems lies in the range of 26–37 °C. It is noted that the block system shows two clear LC transition temperatures whereas the random system only shows a single dominant LC transition although a very weak second LC transition may be present implying less dominant smectic order in LCRBCs. The various mesophases associated with LC transitions are established by X-ray scattering techniques (SAXS and WAXS) and are discussed in the sections below.

## Evolution of phase behavior of TPX75B and TPX75R

Liquid crystalline mesophases depend on molecular shape of the mesogens but are also associated with the strength and position of the polar or polarizable groups within the molecule, polarizability of the molecule, and the presence of chiral centers.^29^ The evolution of hierarchical mesophases in both random and block terpolymer systems is hypothesized to be impacted by π–π stacking and LC interactions. Chromonic mesogens possess liquid-like mobility and retain long-range order at certain concentrations in forming either chromonic N or C phase, which are retained in thermotropic dried films.^30,31^ The intermolecular distance between stacked aggregates in chromonic molecules is known to be 0.34 nm, an indication of π–π stacking arising from aromatic cores which can be observed even at lower concentrations of chromonic systems although it is difficult to ascertain if N or C chromonic phase is produced.^23^ In this effort, we determine the co-existence of π–π stacking of xanthenone, conventional LC interactions and microphase segregation of PEG domains in block and random terpolymers using X-ray scattering techniques and UV analysis of compression molded samples and spin cast films for SEM. Furthermore, the molecular packing, evolution of LC mesophases and influence of terpolymer architectures in thermally annealed films are probed both at room temperature and scanned at different temperatures for the case of temperature controlled SAXS. The concentration of chromonic xanthenone molecules in both block and random terpolymers, TPX75B and TPX75R, respectively, is less than 10 wt%. Thus, while π–π stacking may be noted, the expression of chromonic mesophase (N or C) in this system may be overshadowed by the majority cholesteric mesophases from cholesteric monomers.

### Presence of π–π stacked chromonic mesophase in 75B *vs.* 75R as well as 85B *vs.* 85R

**Fig. 3:**
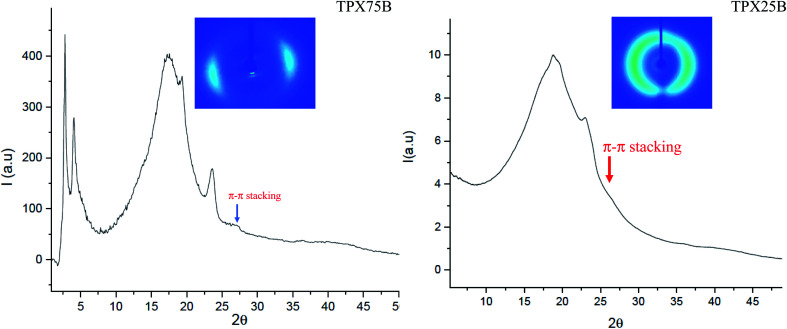
WAXS pattern obtained from compression molded TPX75B and TPX25B where TPX25B has P(NBCh_9_-*b*-NBXan-*b*-NBPEG) with 25/60/15 by composition (wt%). The characteristic peak indicative of face to face π–π stacking appears at 0.34 nm. Inset: 2D WAXS pattern of TPX75B and TPX25B confirm preferentially oriented system.

To fully understand the molecular packing in chromonic molecules incorporated in our terpolymer systems, the presence and distance of π–π chromonic stacking in compression molded films is examined using WAXS (Fig. 3) which reveals a characteristic chromonic face-to-face stacking corresponding to ∼0.34 nm.^32^ Both random and block terpolymers show this peak confirming presence of π–π stacking in both cases. In contrast, previous studies involving LCBBC78 (block copolymers) and LCRBC (random copolymers) that do not contain chromonic molecules did not show corresponding to π–π stacked aromatic cores as expected.^27^

### Presence of smectic polymorphism at room temperature

**Fig. 4:**
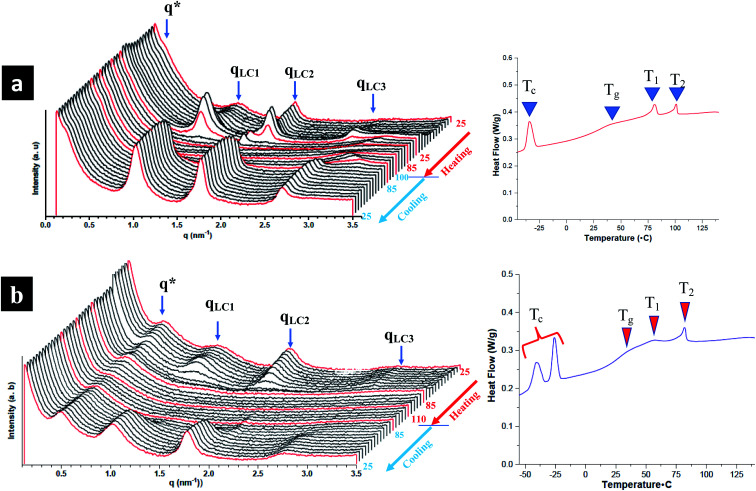
Temperature resolved SAXS and corresponding first cooling cycle from DSC (a) TPX75B and (b) TPX75R from compression molded film. Both samples show co-existence of PEG microphase segregated domains (q*), smectic polymorphism (q_LC1_, q_LC2_ and q_LC3_; *T*_1_) between room temperature and 75 °C and transition from smectic to cholesteric mesophase transition around 75–80 °C (*T*_2_). The morphological evolutions are consistent with results obtained from DSC and PEG microphase segregation is retained even above the LC clearing temperature which is also noted here as *T*_2_.

The influence of LC mesogens, chromonic molecules and PEG brush chains is investigated both at room temperature and on increasing temperature. To investigate smectic polymorphism and microphase segregation in random and block terpolymers, both room and temperature controlled SAXS (TSAXS) are employed. Three types of reflections in TPX75B are determined from SAXS are pertinent to: (i) microphase segregation as indicated by q* (ii) PEG crystallization and (iii) LC order. Presence of uncorrelated reflections imply smectic polymorphism of LC mesogens.^15^ From temperature controlled SAXS, the LC transition to isotropic transition occurs at ∼85 °C (also known as *T*_2_) and there are different levels of intermolecular packing and LC order: PEG domains at ∼14.97 nm are comparable to previous studies while the three LC reflections with domain spacings at 6.10 nm, 3.45 nm and 2.25 nm corresponding to mesophases emanating from NBCh_9_ (Fig. 4). The SmA layer reflections are observed with layer spacings between 3.45 to 6.10 nm. The length of cholesteryl side chain with nine methylene spacers is calculated to be 3.34 nm, and the thickness of smectic bilayer (SmA2) is calculated to be 6.68 nm and this is in agreement with observed results.^15^ This can be attributed to smectic polymorphism common to thermotropic LC polymeric systems where optimal decoupling between backbone and LC mesogen is controlled by type and length of spacer moiety play a vital role. The assignment is based on periodicities in relation to length of the mesogens where smectic monolayer and interdigitated domain spacings are mainly dependent on the spacer length between the backbone and side chains. TPX85B and TPX85R display similar smectic polymorphism and microphase segregation phenomena (Fig. S6 and S7†).

### Presence of microphase segregated PEG domains

The hierarchical arrangement of smectic layers, microphase segregated PEG domains, and morphological evolution of the hierarchical structure are determined using both room and temperature controlled SAXS and the domain sizes of co-existing structures are calculated and tabulated in Table S2.†

All block terpolymers show confined microphase segregated amorphous PEG domain within LC matrix at room temperature. Presence of microphase segregated domains from PEG is noted as q* reflections and higher order reflections are not observed indicating the absence of long-range order.^16,33^ From room temperature SAXS, both TPX75B and TPX85B present microphase segregated domains at ∼28 nm. However, the first q* reflection in the random polymers of TPX75R appears ∼13.2 nm indicating PEG segregated domains. This is in agreement to previously studied systems where random copolymer, LCRBC85, showed PEG segregated domains at ∼12 nm. In TPX75B, TPX75R, TPX85B, PEG domains (i) lack long range order so higher order reflections are not present and (ii) show order-disorder transition that is higher than the LC clearing temperature.

Block copolymers bearing NBCh_9_ and NBMPEG show microphase segregation with higher order peaks where PEG in this system crystallizes into lamella structure.^16^ In contrast, block terpolymers lack higher order peaks and PEG microphase segregation does not order into any familiar morphology. We can attribute this phenomena to the introduction of π–π stacked chromonic units which disrupts the formation of ordered microphase segregated PEG domains. More importantly, the block terpolymers show photonic properties which are absent in the diblock copolymers.

**Fig. 5:**
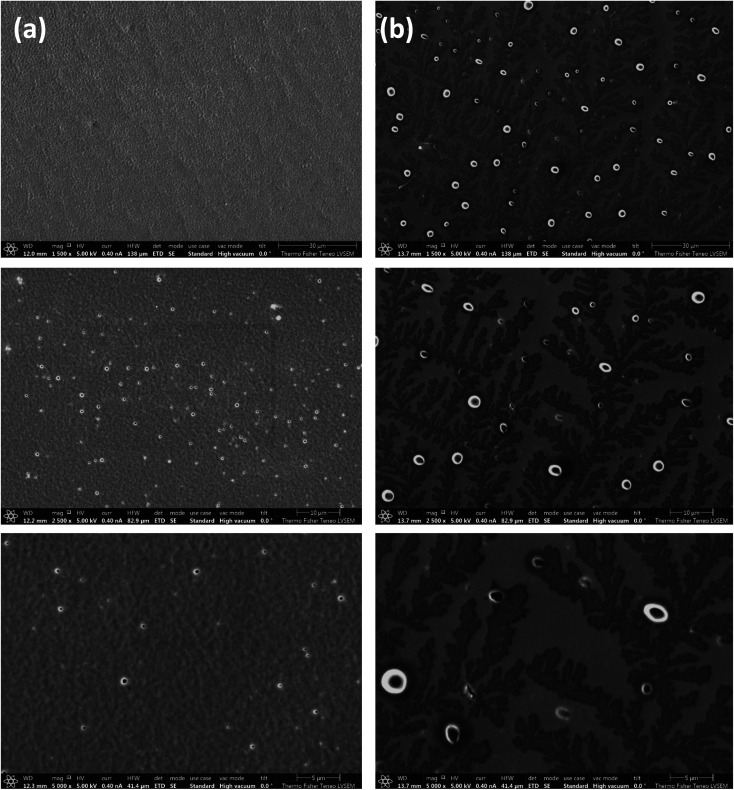
Representative SEM images of TPX75B and TPX75R at different magnification scales (a) cholesteric mesophase showing fingerprint structure of TPX75B (b) spherulitic structures in TPX75R suggesting differences in hierarchical morphology of both systems. Both terpolymers indicate formation of cholesteric mesophases.

SEM samples are prepared by drop casting a thin film of 30% w/v of TPX75B and TPX75R dissolved in THF on a glass slide and allowing the solvent to evaporate. The samples are then annealed in an oven at 84 °C in the cholesteric mesophase for 24 hours. The films are rapidly cooled with cold air once removed from the hot oven to lock mesogens once annealed. Using a tiny blade, the thin film is gently removed from the glass slide and transferred to an SEM stub with the top side of the film facing down on the SEM stub. This sample is then coated with 2.5 nm Au–Pd (80/20) conductive layer before imaging the sample. From SEM images we find that there is a fundamental difference between the block and random terpolymer systems. TPX75B shows conventional fingerprint structures of cholesteric mesophases while TPX75R shows spherulites (Fig. 5).^34,35^ In both cases the block and random terpolymers both form cholesteric mesophases where microphase segregation of amorphous PEG exists. Biopolymers such as chitin, cellulose and chitosan are especially known to form cholesteric spherulites.^36–38^ A case in point is an observation made by Zhiyu and coworkers where TiO_2_ nanorods when dissolved in solution form lyotropic solutions exhibit cholesteric spherulites.^39^

The morphological evolution of TPX75B with temperature shows that at room temperature π–π chromonic stacking, smectic mesophases and PEG microphase segregation co-exist and evolves into π–π chromonic stacking with cholesteric mesophase and PEG microphase segregation. This enables the expression of 1D photonic properties that will be described in the next section. In previous studies, block copolymers bearing only NBCh_9_ and NBMPEG do not show any cholesteric mesophase or resultant 1D photonic properties. These LCBBCs incorporate microphase segregation of PEG, PEG crystallization and LC order where PEG cylinders form within the cholesteric majority matrix and effectively prevents 1D photonic features from being expressed. For example, in LCBBC78, PEG cylinders form in a majority LC matrix and as such disrupt the expression of cholesteric optical properties. Additionally, within these cylinders semicrystalline PEG domains are present as confirmed by SAXS, DSC and WAXS studies. In contrast, when a chromonic block bearing a xanthenone unit is introduced, the interface between the domains in TPX75B is modified and allows the cholesteric features to be expressed by disrupting formation of PEG crystalline domains. This is the first time, confined microphase segregated PEG domains co-existing with cholesteric LC matrix in the block systems has been observed.

## Optical properties

Cholesteric liquid crystalline polymers are characterized with continuous twisted arrangements of chiral mesogens (helical cholesteric mesophase, N*) featuring 1D photonic properties that can be tuned by use of a stimulus such as heat, light or temperature. In our previous publications, we showed that cholesteric polymers can be tuned to reflect different wavelengths (colors) by use of temperature.^27,33^ Furthermore, incorporation of other LC monomers in the polymer framework particularly azobenzene and other achiral LCs has an influence on the optical properties and these materials show red or blue shifts depending on LC–LC interactions. In this work azobenzene is substituted for a chromonic monomer and the cholesteric–chromonic mesophase interactions disrupts the cholesteric pitch length of these terpolymers. Interestingly, all the random and block terpolymer systems prepared here composed of chromonic–cholesteric interactions show blue, green or blue green reflections with maximum wavelength of around 475 nm.

**Fig. 6:**
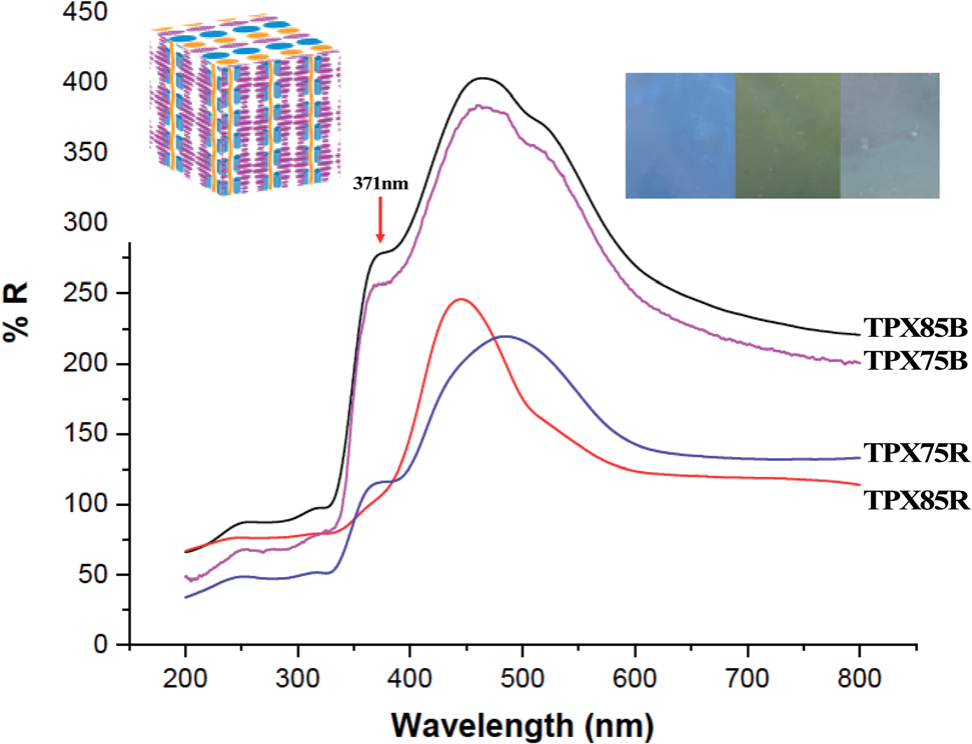
UV Reflectance data obtained from TPX polymer films samples compression molded at distinctive temperatures depending on the liquid crystalline transition temperatures. The transition at 371 nm is indicative of π–π stacking characteristic of chromonic mesophases, which manifests as π–π* transition in the reflection spectra.

Solid polymer powders are compression molded and annealed between two Kapton films at with reference to *T*_1_ or *T*_2_ for UV-vis measurements. The samples are then rapidly quenched to room temperature to kinetically trap the cholesteric mesophase using cold air to a temperature below *T*_g_ to preserve the cholesteric mesophase while retaining the reflected color.^40,41^ Cholesteric materials are known as 1D bandgap materials with selective light reflection at wavelengths corresponding to the product of the helical pitch and the average refractive index of the medium.^42^ Existence of a cholesteric mesophase above ∼81 °C is supported by UV-vis studies under reflectance mode. In both block terpolymers and random terpolymer films, we observe a peak at 371 nm which is a further indication that π–π* transition that occurs in this system from stacked chromonic molecules, Fig. 6.^43,44^ Thus in all these samples π–π chromonic samples co-exist with cholesteric mesophase and in three samples, π–π stacking, PEG microphase segregation and cholesteric mesophase co-exist.

In block copolymers containing chiral LCs, microphase segregated domains and high surface to volume ratio at the intermaterial dividing surface (IMDS) induce cholesteryl mesogens to homogeneously anchor at the interface. This generally promotes smectic to smectic transitions leading up to LC clearing transition and cholesteric mesophase cannot be produced or stabilized.^16^ However, in the current LC block terpolymers containing chromonic molecules, microphase segregated PEG do not form semicrystalline lamella/cylinders within the cholesteric matrix as supported by SAXS and as such hinders such smectic to smectic to LC clearing transitions. We hypothesize that this in effect can change the anchoring at the IMDS, a modification promoted by chromonic xanthenone. This modification of the interface using π–π using chromonic molecules allows for smectic to cholesteric to LC clearings transitions can be confirmed by thermal, X-ray and UV characterization. Furthermore, xanthenone molecules in random and block terpolymer system acts to impede formation of longer pitch of the cholesteric mesophase and red-shift in the visible spectra. Finally, π–π* transition arising from π–π stacking of chromonic molecules (xanthenone) manifests when UV-vis reflectance measurements are made.^43^

Due to the presence of π–π stacking, the block and random terpolymer show similar morphological features for 75% composition and this manifests into similar optical behavior, that is, π–π stacking manifesting as π–π* reflections and the reflection from the cholesteric phase. We do not see any major shift in reflection that is expected from convention LC–LC interactions, in this case chromonic–cholesteric interactions.^9^ We also do not see any impact of microphase segregation on blue shift that has been observed in other LCRBCs. Thus, by introducing chromonic molecules and π–π interactions, block copolymer systems lose the ability to form highly ordered microphase segregated structures without curved interfaces^45^ and instead are destined to self-assemble into microphase segregated structures without long-range order in conjunction with curved mesophases similar to the random systems and thus show optical properties reminiscent of random systems, for the first time.

This study presents the first examples of homopolymers and terpolymers containing chromonic molecules that can be dissolved in organic solvents, form good films and can overcome some of the surface effects observed in chromonic systems that are soluble in aqueous media.^46–48^ While the homopolymer containing xanthenone does not form chromonic mesophases, both random and block terpolymers self-assemble into chromonic mesophases in the presence of cholesteryl LC molecules. The ability to produce chromonic mesophases from within polymer films, as opposed to just small molecular solution assembly, is advantageous as the chemical, thermal and mechanical stability of these systems can be tailored for a variety of applications.^46,49,50^

## Conclusions

We report the synthesis and characterization of random and block terpolymers by ROMP of NBCh_9_, NBXan, and NBMPEG. The impact of chromonic molecules on chromonic–LC interactions, hierarchical self-assembly, and thermal properties in block and random terpolymers are investigated. The interface between the blocks in block terpolymers is modified and manifests into the co-existence of microphase segregated PEG domains, chromonic π–π interactions and smectic polymorphism at room temperature. This evolves into microphase segregated PEG domains, chromonic π–π interactions and cholesteric mesophase at 75 °C and above. This observation in block terpolymers is contrary to previous LCBCPs and LCBBC where cholesteric mesophase has never been noted. The presence of π–π chromonic interactions co-existing with cholesteric mesophases in the block and random terpolymers manifests as visible reflections from π–π* transitions and cholesteric mesophase in the optical spectra of these polymers. In this work, we show that using supramolecular chromonic π–π interactions single component liquid crystalline block copolymers will present morphology and optical properties similar to nanoscale mesophases resulting in liquid crystalline random copolymer and terpolymer architectures.

## Conflicts of interest

There are no conflicts of interest to declare.

## Supplementary Material

RA-011-D1RA00899D-s001
