# Supplementary material for: Cholesteric mesophase based 1D photonic materials from self-assembly of liquid crystalline block and random terpolymers containing chromonic molecules
Source: RSC Adv. 2021 Apr 20;11(24):14615–23. doi: 10.1039/d1ra00899d (PMC8697838; doi:10.1039/d1ra00899d)
Supplement: RA-011-D1RA00899D-s001 [file RA-011-D1RA00899D-s001.pdf]

Electronic Supplementary Material (ESI) for RSC Advances.
This journal is © The Royal Society of Chemistry 2021

Electronic Supporting Information

Cholesteric mesophase based 1D photonic materials from self-assembly of liquid crystalline block and random terpolymers containing chromonic molecules

Reuben Bosire[†], Dennis Ndaya[†], Rajeswari Kasi[†,§]

[†]Department of Chemistry, University of Connecticut, Storrs, CT 06269 (USA)

[§] Polymer Program, Institute of Material Science, University of Connecticut, Storrs, CT 06269 (USA)

1. Materials

Norbornene-2-carboxylic acid (mixture of endo and exo, 98%), Second generation Grubbs catalyst, 3-hydroxy-9H-9-xanthenone, and 4-dimethylaminopyridine are purchased from Sigma-Aldrich and used without further purification. Modified Grubbs catalyst second generation (H2IMes)(pyr)2(Cl)2RuCHPh (mG2)[1] is synthesized using a reported procedure. Dry methylene chloride (CH_2Cl_2, 99.8%), anhydrous tetrahydrofuran (THF, 99.9%), anhydrous dimethylformamide (DMF), oxalyl chloride (98%), ethyl vinyl ether (EVE, 99%), and pentane (99%) are obtained from Acros Organics while 12-bromo-1-dodecanol is obtained from TCI America. N-(3-Dimethylaminopropyl)-N'-ethylcarbodiimide hydrochloride (EDC) 98% is obtained from Proteochem.

2. Characterization

^{1} H NMR spectroscopy is conducted on Bruker DMX 500 MHz NMR spectrometer with $CDCl_3$ as the lock solvent at room temperature. ^{1}H NMR chemical shifts are delineated in ppm downfield from TMS. The molecular weight of NBPLA is determined by gel permeation chromatography (GPC) using a Waters 1515 coupled with a PL-ELS1000 evaporative light scattering (ELS) detector and a Waters 2487 dual-wavelength absorbance UV-Vis detector with tetrahydrofuran (THF) as eluent and polystyrene (PS) standards for constructing a conventional calibration curve. Differential scanning calorimetry (DSC) is conducted on TA-2920 instrument (Q-200 series) calibrated with an Indium standard. The amount of sample used is 5-10 mg and the scanning rate is 10 °C/min. Phase transition temperatures are determined by the first cooling cycle using Universal Analysis software. Thermogravimetric analysis is performed using 5-10 mg of the sample on a TGA Q500 1732 for analysis of thermal properties at 20° C/min under Nitrogen. Scanning electron microscope (SEM) images of all samples are recorded using an FEI Teneo LVSEM equipped with an ETD detector with an accelerating voltage 15 kV at UCONN/FEI Center for Advanced Microscopy and Materials Analysis (CAMMA). The film samples are prepared by compression molding and sputter coating with a thin gold conductive layer before imaging. UV spectroscopic data is obtained from Shimadzu UV-Vis Spectrometer (UV-2450) in reflectance mode with a wavelength range of 200 -1000 nm. The free-standing films (~ 0.3 mm in thickness) are prepared by annealing the solid samples (100- 200 mg) in the temperature range of 85- 104°C between the polyimide Kapton films at the liquid crystalline transition temperature for

~ 1 hour. The liquid crystalline transition phase is preserved by rapidly cooling the sample to room temperature ($T < T_g$) using cold compressed air.

Small angle X-ray diffraction experiments are carried out on a pin-hole collimated Rigaku SMAX3000 instrument configured with CuKα radiation (1.542 Å) produced by a micro-focus source. The beam diameter on the sample plane is 1 mm and the scattered intensity is recorded on a gas-wire electronic area (2D) detector. The area detector has a resolution of 1024 × 1024 pixels, located at ~80 cm from the sample center permitting access of scattering vectors ranging from 0.015 to 0.35 $Å^{-1}$. Silver behenate (d-spacing=58.38 Å) is used to calibrate the SAXS diffraction patterns. WAXS is performed on an Oxford Diffraction XCalibur PX Ultra (transmission mode) diffractometer with an Onyx detector (Cu Ka radiation 1.542 A, double mirror focusing, 45 kV and 45 mA).

Monomer Synthesis

The synthesis of monomers NBCh9 and NBMPEG has been reported in previous publications.[1, 2] A new monomer, 12-((9-oxo-9*H*-xanthen-3-yl)oxy)dodecyl(1*R*,4*R*)-bicyclo[2.2.1]hept-5-ene-2-carboxylate, abbreviated as NBXan is synthesized following a newly developed protocol as outlined in in Scheme S1. In a typical synthesis, 3-hydroxy-9H-9-xanthenone (4 g, 188.63 mmol), 12-bromododecanol (7.51 g, 28.3 mmol) and potassium carbonate (5.21g, 37.73 mmol) are transferred to a round-bottomed flask along with a magnetic stir bar, followed by addition of 60 mL of DMF, and sealed with a rubber septum. The reaction mixture is purged with nitrogen for 10 min, and then stirred in oil bath for 48 h at 95 °C. The reaction mixture is cooled down to room temperature and diluted with 100 mL of CH_2Cl_2. The mixture is washed with 200 mL of deionized water twice, and treated with brine (100 mL) to remove DMF. Organic layer is separated from the aqueous layer, collected and dried with $MgSO_4$. The crude product is purified by recrystallization using 50 mL of ethanol resulting in 6.9 g of compound A, yield = 76%.

Compound A (5 g, 126.09 mmol) is dissolved in minimum chloroform and transferred dropwise to a vigorously stirring mixture of 5-norbonene-2-carboxylic acid (1.92 g, 138.70 mmol), EDC (2.66 g, 138.70 mmol) and DMAP (0.077 g, 12.2 mmol) in 25 mL of chloroform at 0 ˚C in a round bottom flask. After 30 minutes of stirring at 0 ˚C, the reaction is allowed to warm to room temperature and stirred for twelve hours. After twelve hours of reaction, the mixture is diluted with

chloroform and filtered. The filtered mixture is then transferred to a separating funnel, washed with distilled water (2 x 100 mL). The organic phase is dried over $MgSO_4$ and resultant product is filtered, concentrated then purified on a silica column eluting with 3:2 ethyl acetate/hexane. The purity of this newly synthesized monomer (NBXan) is confirmed by NMR and LC-MS with results in agreement with theoretically determined values.

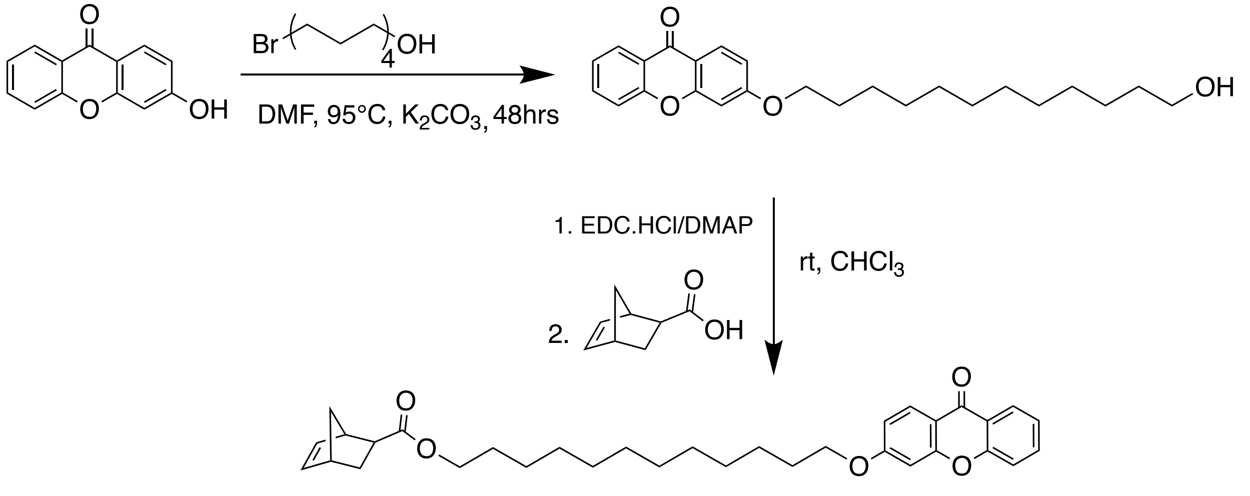

12-((9-oxo-9H-xanthen-3-yl)oxy)dodecyl(1R,4R)-bicyclo [2.2.1] hept-5-ene-2-carboxylate

Scheme S1: Synthesis of NBXan (12-((9-oxo-9*H*-xanthen-3-yl)oxy)dodecyl(1*R*,4*R*)-bicyclo [2.2.1] hept-5-ene-2-carboxylate).

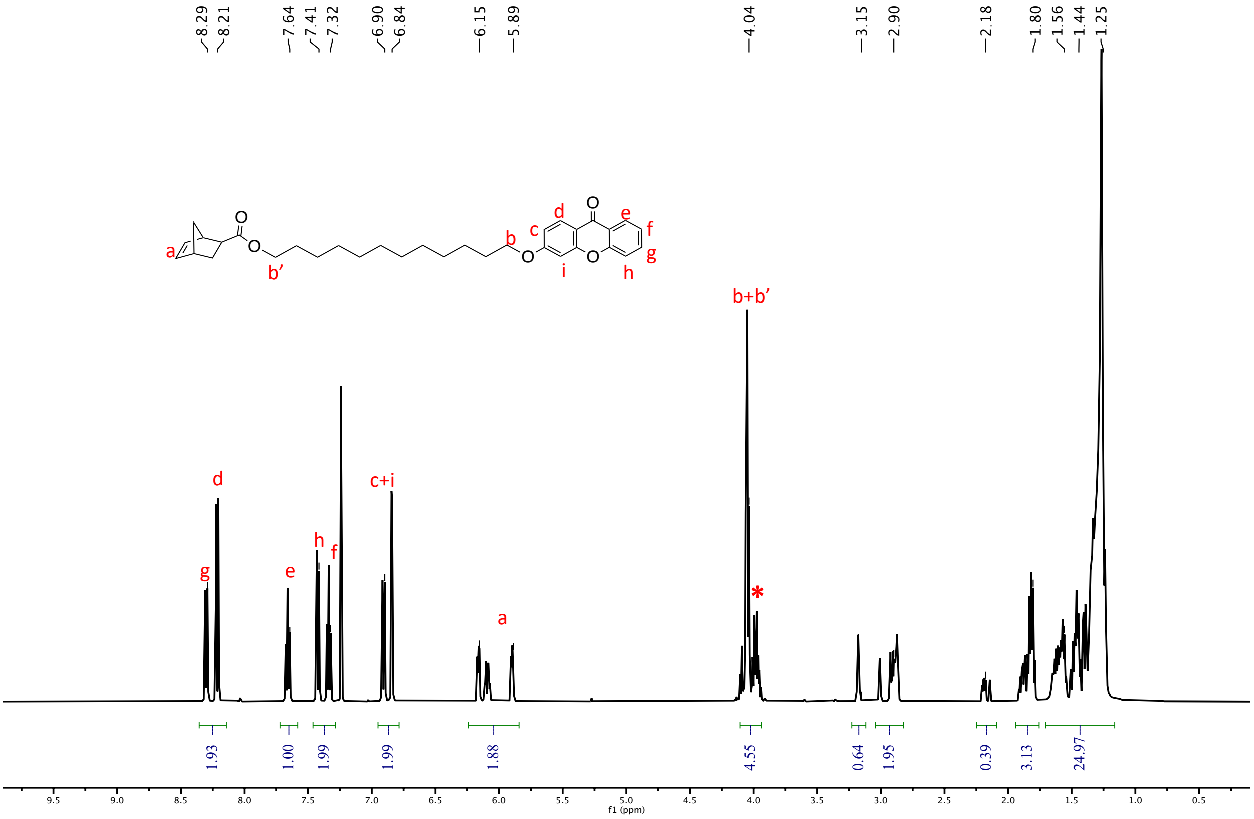

Figure S1: Representative ^{1}H NMR for NBXan at room temperature in $CDCl_3$. The * represents contribution from ester peak from ethyl acetate used as part of eluent solvent during column purification.

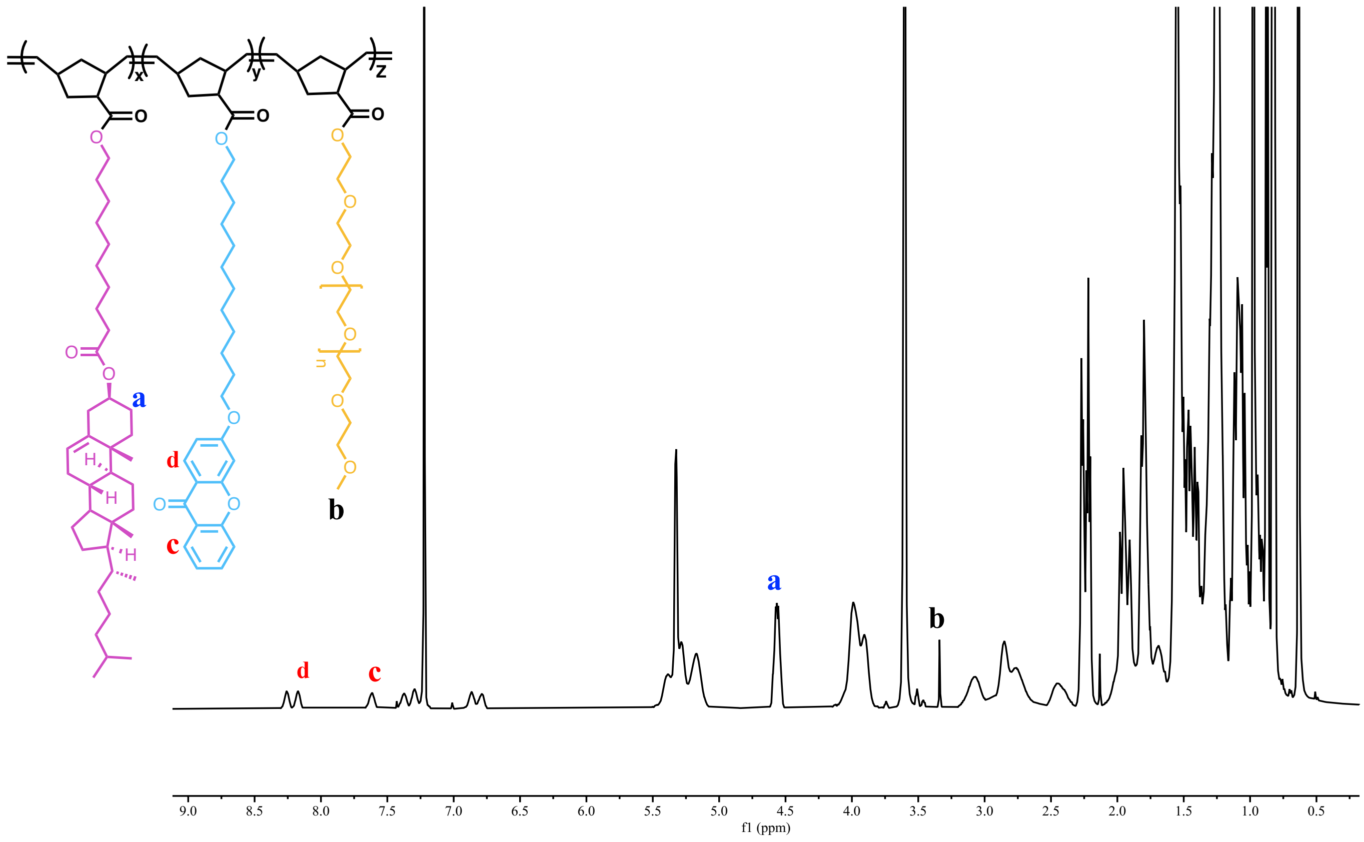

Figure S2: Representative ^{1}H NMR for TPX75 random terpolymer at room temperature in $CDCl_3$

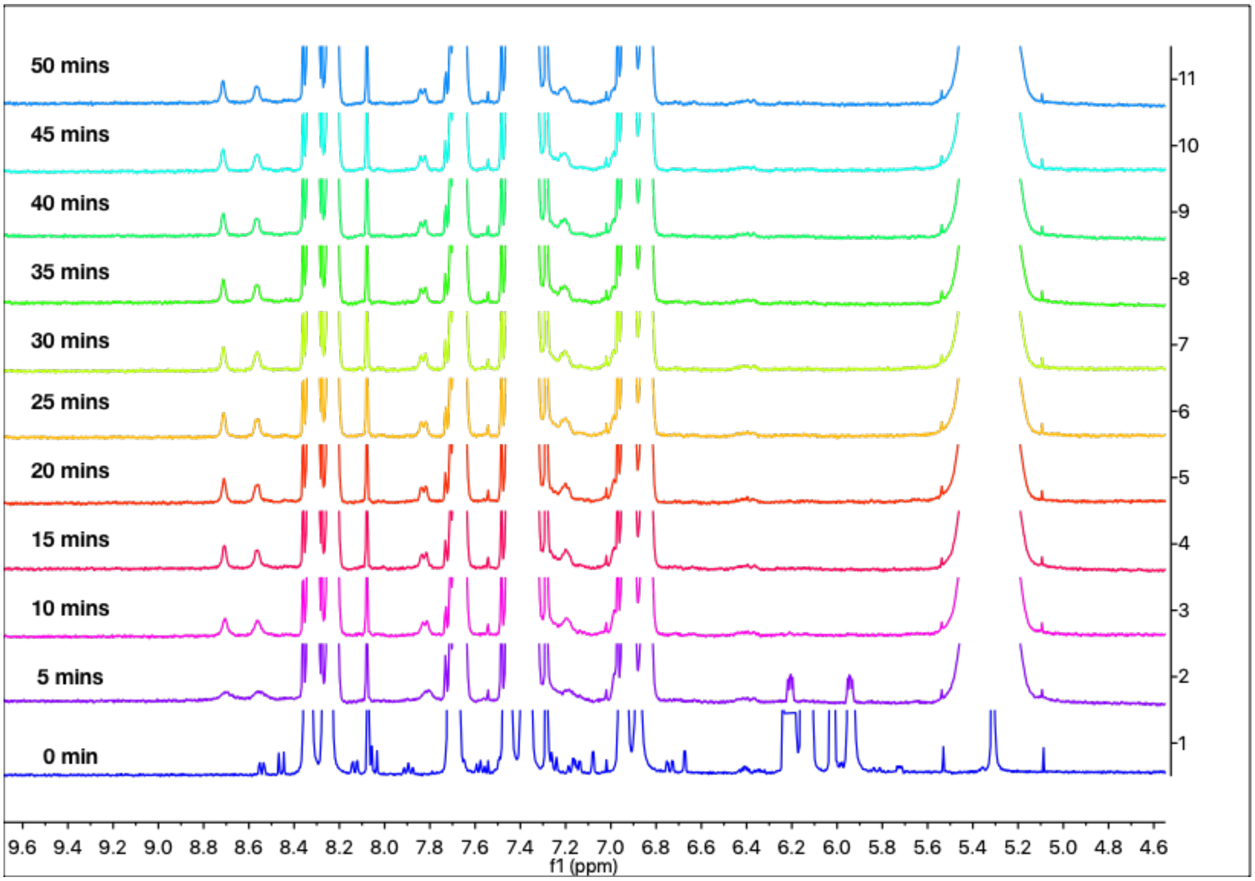

Figure S3: ^{1}H NMR study of polymerization kinetics of newly synthesized monomer, NBXan. The olefinic peaks at ~5.9 – 6.3 ppm (from norbornene backbone) are monitored as they shift (disappear) upfield to 5.3 ppm as the polymerization progresses and we determine that >93% conversion is achieved by the 5th minute.

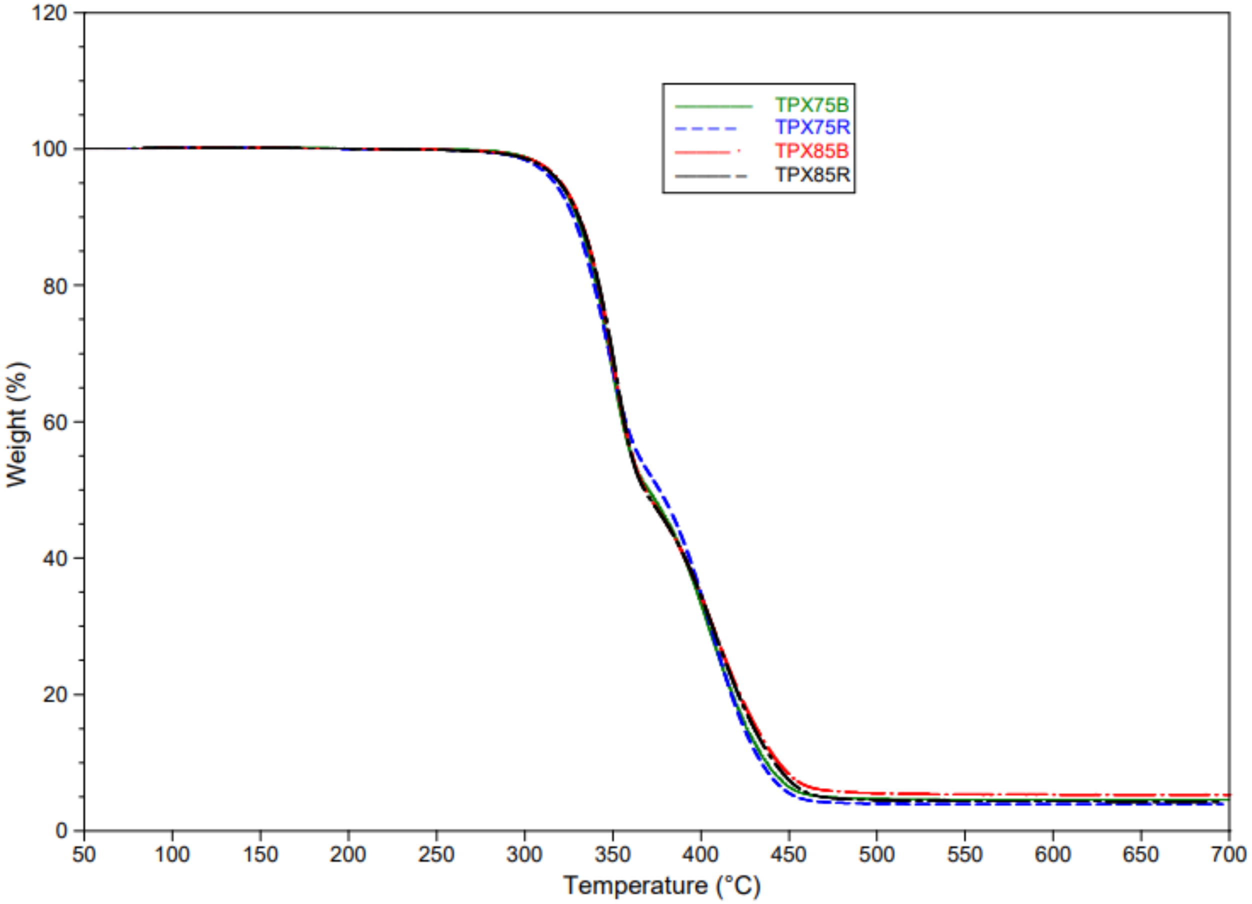

Figure S4: TGA traces showing thermal stability of block and random terpolymers. At 300 ºC, only 1.4% of both block and random terpolymers has degraded.

Table S1: Summary of thermal properties from block and random terpolymers

Entry	*T_c °C*	*T_g (°C)*	*T_1,°C (ΔH J/g)*	*T_2,°C (ΔH J/g)*
**TPX75B**	-34.5	31.4	80.7 (1.2)	100.9 (0.8)
**TPX85B**	-33.7	30.3	80.9 (1.2)	100.7 (0.7)
**TPX75R**	-21.8-37.9	28.6	83.54 (1.0)	
**TPX85R**	-23.6-41.6	37.2	87.7(1.1)	

All transition temperatures were determined from DSC first cooling cycle at a scan rate of 10°C/min. ΔH values (where applicable) correspond to T_c, T_1,T_2 respectively.

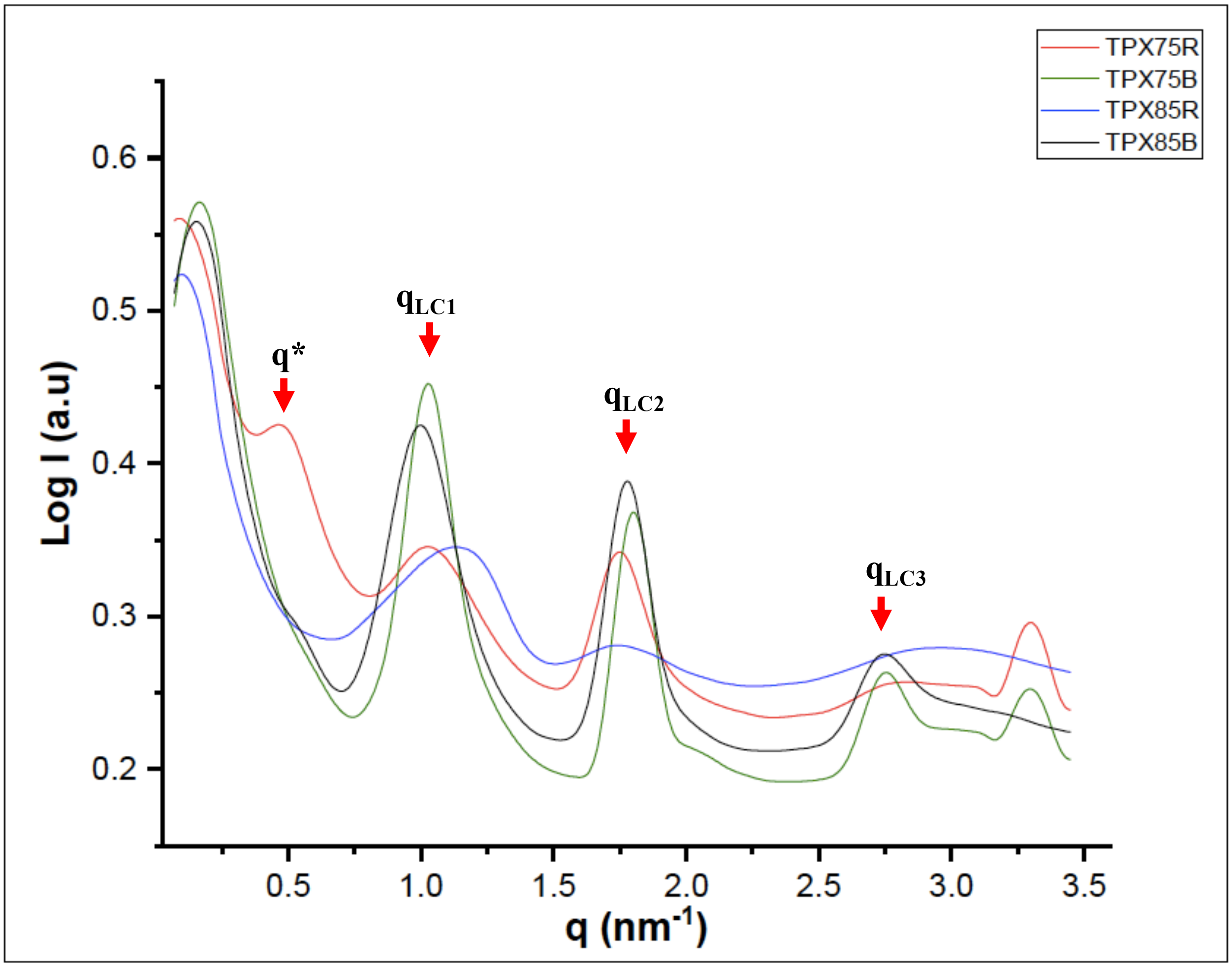

Figure S5: Room temperature 1D-SAXS for block and random terpolymers indicating presence of smectic polymorphism and confined microphase segregated amorphous PEG domains within LC matrix at room temperature.

Table S2: Domain spacing values for block and random terpolymers as determined by SAXS at room temperature.

Polymer	q*(d-spacing, nm)	d_1 nm	d_2 nm	d_3 nm	d_4 nm	d_5 nm
TPX75B	28.56	6.08	3.94	3.51	2.29	1.97
TPX85B	30.63	6.13	3.49	2.29		
TPX75R	13.22	6.18	3.56	2.28		
TPX85R	-	6.23	3.55	2.32		

Note: q* is from microphase segregation of PEG and d_1-d_5 comes from LC smectic polymorphism.

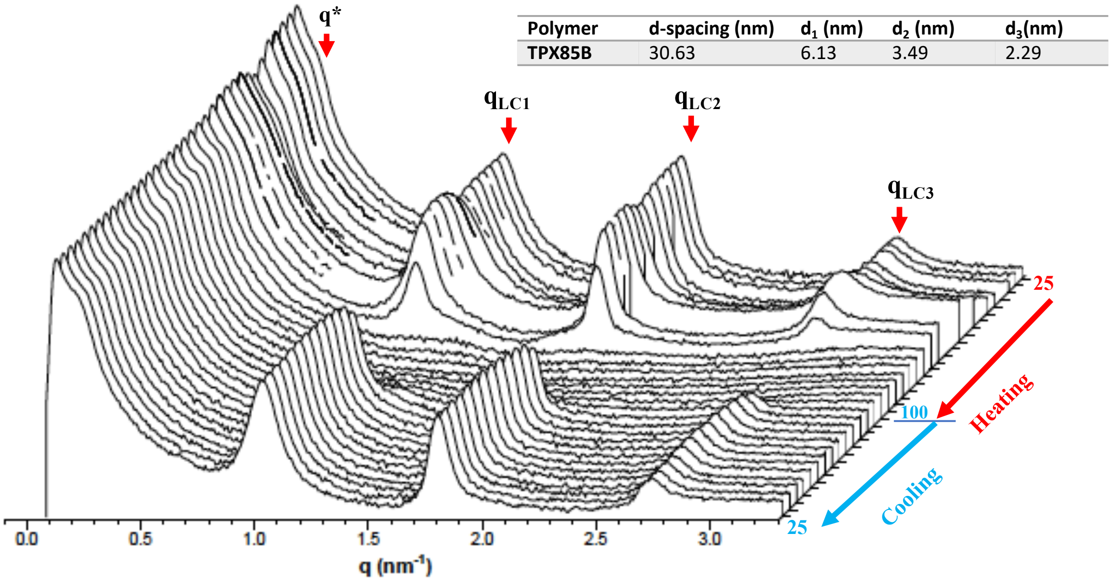

Polymer	d-spacing (nm)	d_1 (nm)	d_2 (nm)	d_3(nm)
TPX85B	30.63	6.13	3.49	2.29

Figure S6: Temperature controlled SAXS for TPX85B. The transition from smectic to cholesteric to isotropic can be seen as the peak reflections (q_{LC1}, q_{LC2} and q_{LC3}) disappear at ~85^{0}C. The domain sizes are in nm. Microphase segregation of amorphous PEG domains occurs at ~30 nm (q* reflection) for LCBBC terpolymers and is maintained throughout the heating and cooling cycle.

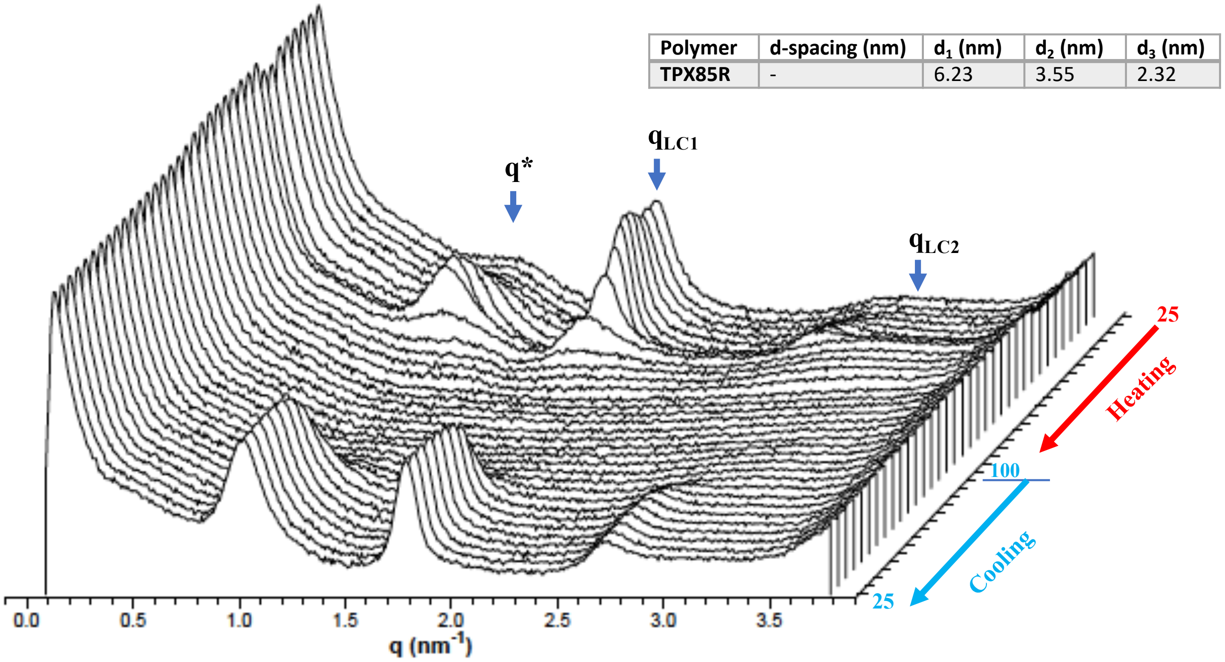

Polymer	d-spacing (nm)	d_1 (nm)	d_2 (nm)	d_3 (nm)
TPX85R	-	6.23	3.55	2.32

Figure S7: Temperature controlled SAXS for TPX85R. The domain sizes are in nm. With the random architecture there is no microphase segregation of PEG brushes. Microphase segregated peak (q*) of amorphous PEG domains which occurs at ~13 nm for LCRBC terpolymers is absent in this terpolymer.

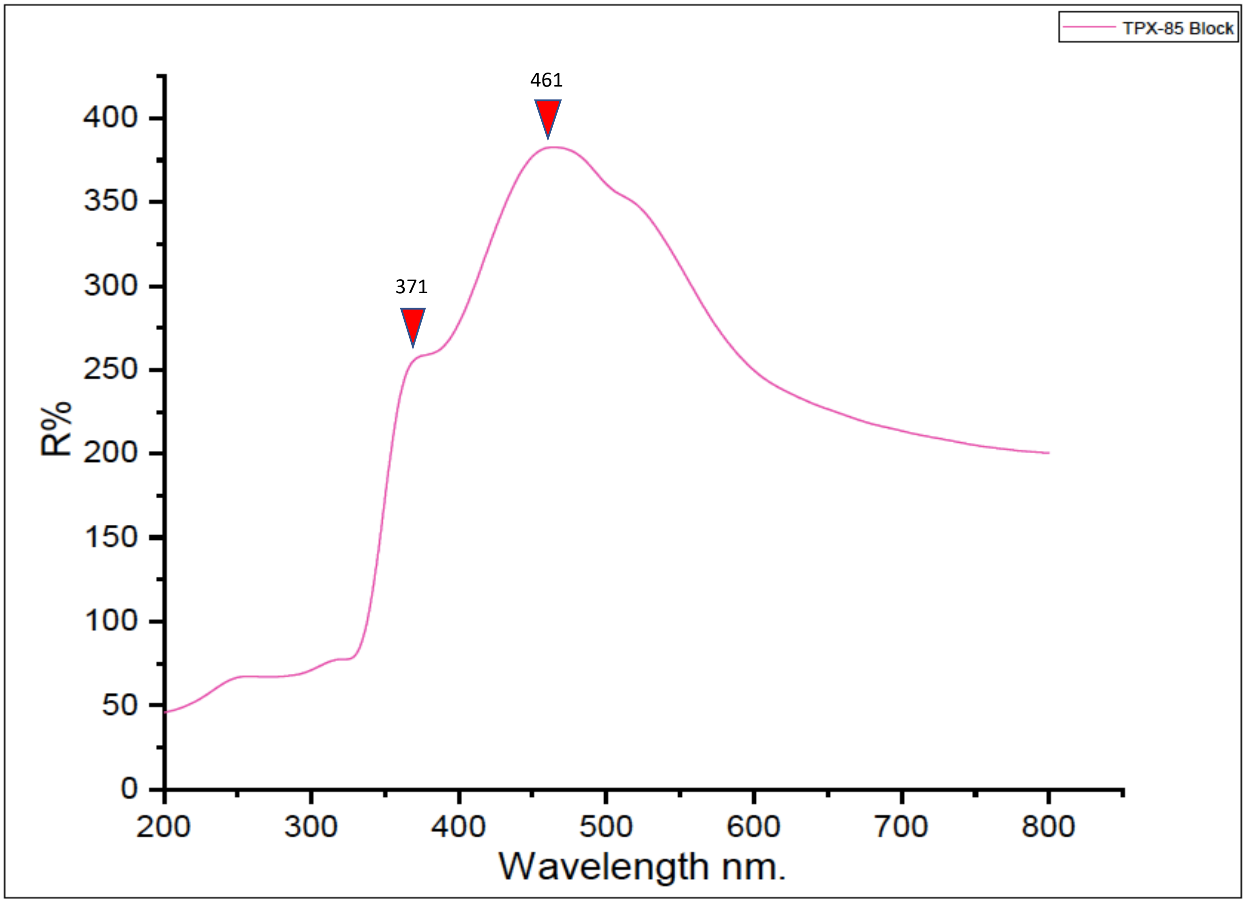

Figure S8: Reflectance from TPX85B showing effect of interaction of cholesteric-chromonic mesophase. The main reflection at 461 nm corresponds to cholesteric mesophase whereas the reflection at 371 nm comes from π-π stacking of xanthenone chromonic molecules.[3]

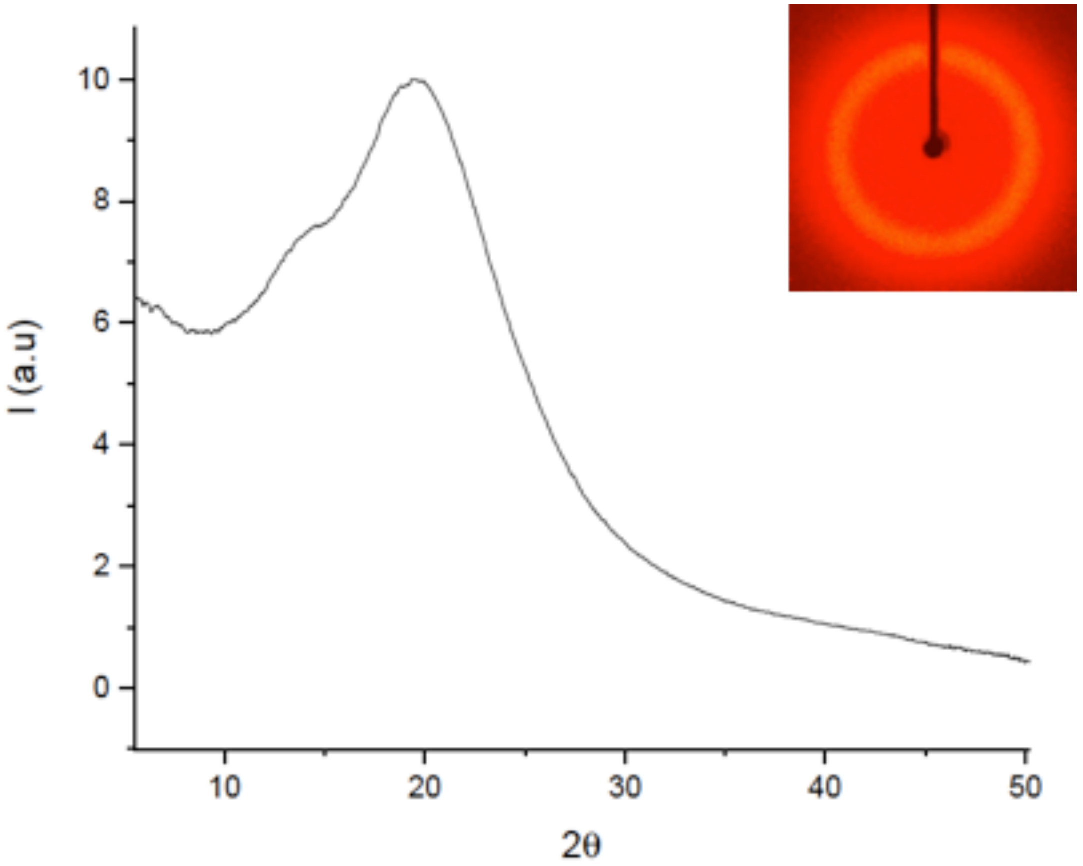

Figure S9: WAXS pattern obtained from compression molded xanthenone homopolymer P(NBXan). From WAXS, π-π stacking is not observed in the homopolymer. Thus, the presence of LC-LC interactions between cholesteryl and xanthenone units in the block and random terpolymer containing chromonic molecules is essential to also produce π-π stacking.

References

1. Zhou, Y.; Briand, V. A.; Sharma, N.; Ahn, S.-k.; Kasi, R. M., Polymers comprising cholesterol: synthesis, self-assembly, and applications. *Materials* **2009,** *2* (2), 636-660.
2. Deshmukh, P.; Ahn, S.-k.; Gopinadhan, M.; Osuji, C. O.; Kasi, R. M., Hierarchically Self-Assembled Photonic Materials from Liquid Crystalline Random Brush Copolymers. *Macromolecules* **2013,** *46* (11), 4558-4566.
3. Veerabhadraswamy, B. N.; Rao, D. S.; Yelamaggad, C. V., Stable ferroelectric liquid crystals derived from salicylaldimine-core. *J Phys Chem B* **2015,** *119* (12), 4539-51.
